# Health impacts of lifestyle and ambient air pollution patterns on all-cause mortality: a UK Biobank cohort study

**DOI:** 10.1186/s12889-024-19183-5

**Published:** 2024-06-25

**Authors:** Lining Pu, Yongbin Zhu, Xiaojuan Shi, Huihui Wang, Degong Pan, Xiaoxue He, Xue Zhang, Liqun Wang, Xiaojuan Liu, Shulan He, Xian Sun, Jiangping Li

**Affiliations:** 1 Department of Epidemiology and Health Statistics, School of Public Health, Ningxia Medical University, Ningxia Hui Autonomous Region, Yinchuan, 750004 China; 2 Key Laboratory of Environmental Factors and Chronic Disease Control, Ningxia Medical University, Ningxia Hui Autonomous Region, Yinchuan, 750004 China

**Keywords:** Air pollution, Lifestyle, All-cause mortality, Latent profile analysis

## Abstract

**Background:**

Extensive evidence indicates that both lifestyle factors and air pollution are strongly associated with all-cause mortality. However, little studies in this field have integrated these two factors in order to examine their relationship with mortality and explore potential interactions.

**Methods:**

A cohort of 271,075 participants from the UK Biobank underwent analysis. Lifestyles in terms of five modifiable factors, namely smoking, alcohol consumption, physical activity, diet, and sleep quality, were classified as unhealthy (0–1 score), general (2–3 score), and healthy (4–5 score). Air pollution, including particle matter with a diameter ≤ 2.5 μm (PM_2.5_), particulate matter with a diameter ≤ 10 μm (PM_10_), particulate matter with a diameter 2.5–10 μm (PM_2.5−10_), nitrogen dioxide (NO_2_), and nitrogen oxides (NO_x_), was divided into three levels (high, moderate, and low) using Latent Profile Analysis (LPA). Cox proportional hazard regression analysis was performed to examine the links between lifestyle, air pollution, and all-cause mortality before and after adjustment for potential confounders. Restricted cubic spline curves featuring three knots were incorporated to determine nonlinear relationships. The robustness of the findings was assessed via subgroup and sensitivity analyses.

**Results:**

With unhealthy lifestyles have a significantly enhanced risk of death compared to people with general lifestyles (HR = 1.315, 95% CI, 1.277–1.355), while people with healthy lifestyles have a significantly lower risk of death (HR = 0.821, 95% CI, 0.785–0.858). Notably, the difference in risk between moderate air pollution and mortality risk remained insignificant (HR = 0.993, 95% CI, 0.945–1.044). High air pollution, on the other hand, was independently linked to increased mortality risk as compared to low air pollution (HR = 1.162, 95% CI, 1.124–1.201). The relationship between NO_x_, PM_10_, and PM_2.5−10_ and all-cause mortality was found to be nonlinear (*p* for nonlinearity < 0.05). Furthermore, no significant interaction was identified between lifestyle and air pollution with respect to all-cause mortality.

**Conclusions:**

Exposure to ambient air pollution elevated the likelihood of mortality from any cause, which was impacted by individual lifestyles. To alleviate this hazard, it is crucial for authorities to escalate environmental interventions, while individuals should proactively embrace and sustain healthy lifestyles.

**Supplementary Information:**

The online version contains supplementary material available at 10.1186/s12889-024-19183-5.

## Background

The adoption of healthy lifestyles has been widely recognized as crucial to mitigating the economic and medical implications associated with various diseases [1, 2]. According to the American Heart Association, lifestyle factors mainly included physical activity, nicotine exposure, sleep health, Body Mass Index (BMI), diet habits, and more [3]. Previous studies have revealed that smoking, physical inactivity, poor diet, and heavy alcohol consumption result in 60% of premature mortality and reduce life expectancy by 7.4–17.9 years [4, 5]. Furthermore, it has been demonstrated that unhealthy lifestyles significantly increase the risk of coronary heart disease, hypertension, diabetes, cancer, and cardiovascular disease [6]. Therefore, it is imperative to actively guide and encourage individuals to make changes to unhealthy lifestyles in order to improve the overall quality of human life.

Simultaneously, ambient air pollution has been closely linked to human health and has been identified as a contributor to the global burden of disease, as outlined in the 2019 Global Health Guidelines [7]. It has been estimated that in 2018, 6.0% of all global deaths were caused by ambient air pollution [8]. Studies have revealed that long-term exposure to air pollutants is associated with an increased risk of all-cause and cause-specific deaths [9–11]. However, previous studies have mainly focused on examining the relationship between a single air pollutant and mortality [12, 13]. It is crucial to note that in reality, humans are often exposed to multiple air pollutants simultaneously, making it essential for us to consider the combined effects of multiple pollutants on mortality. Latent profile analysis (LPA) is a flexible, model-based clustering technique that can identify subtypes of homogeneous potential classes or subgroups within a large heterogeneous population [14]. To the best of our knowledge, recent studies using LPA to classify individuals based on air pollution are scarce.

Extensive research has demonstrated an association between mixtures of pollutants and all-cause mortality [11, 15, 16], while other studies have established a link between lifestyle factors and mortality rates [17–21]. Nevertheless, there remain knowledge gaps that need to be addressed. Firstly, longitudinal studies that examine the combined relationship between lifestyles, air pollution, and all-cause mortality are insufficient. Secondly, research on the interactions of lifestyles and air pollution with health outcomes is inadequate. Furthermore, it remains unclear whether these findings are consistent across subgroups of different age, gender, ethnicity, and education level.

We conducted a population-based prospective cohort study to investigate the potential correlation between air pollution, comprising particle matter (PM_2.5_, PM_10_, PM_2.5−10_), nitrogen dioxide (NO_2_), and nitrogen oxides (NO_x_), and various lifestyle factors, including smoking, alcohol consumption, diet, sleep, and physical activity, in relation to all-cause mortality risk. Furthermore, we aimed to explore whether this relationship was modified by different subgroups.

## Methods

### Study design and population

This study was conducted utilizing the UK Biobank, which received approval from the North West Multicenter Research Ethics Committee. The UK Biobank is a comprehensive biomedical database and research resource containing in-depth genetic and health information from half a million UK participants. The participants were recruited from 22 centers in England, Wales, and Scotland between 2006 and 2010, and were aged between 37 and 73 [22]. The health information provided by the participants was collected through touchscreen questionnaires, verbal interviews, physical measures, and biological samples.

**Fig. 1:**
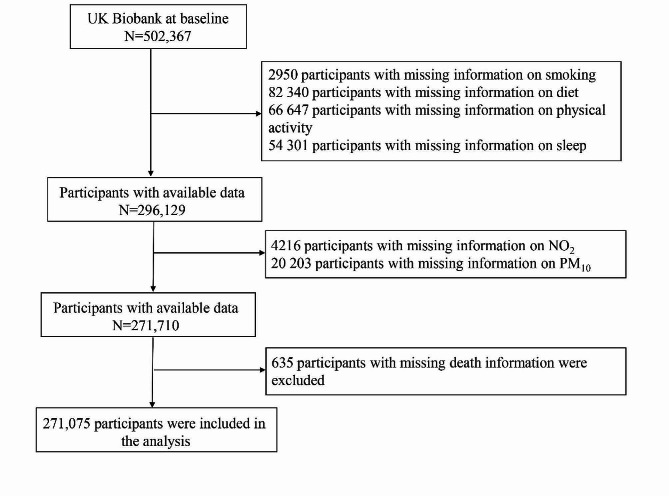
Flow chart of creation of the dataset

For this study, participants with missing information on lifestyle factors such as smoking (*N* = 2950), diet (*N* = 82,340), physical activity (*N* = 66,647), and sleep quality (*N* = 54,301) were excluded. After these exclusions, the remaining number of participants was 296,129. Additionally, participants lacking air pollution data, such as NO_2_ (*N* = 4216) and PM_10_ (*N* = 20,203), were also excluded. Finally, participants without recorded death information (*N* = 635) were excluded from the analysis. The final analysis included a total of 271,075 participants (Fig. 1).

### Assessment of Lifestyle

At baseline, we collected lifestyle information and evaluated lifestyle patterns based on five modifiable factors: smoking, alcohol consumption, physical activity, diet, and sleep quality, in view of the previous studies [23–26]. Smoking was categorized as never smoked or current/previous smoker. Never smoking was considered a healthy lifestyle. For alcohol consumption, we calculated the daily intake of pure alcohol based on the average number of alcoholic drinks consumed per week, the number of grams of alcohol in each drink, then dividing it by seven. The drinks included red wine, white wine, beer, spirits, and fortified wine. Those who consumed less than 30 g of pure alcohol per day for males and less than 20 g for females were considered to adhere to a healthy lifestyle [27], while those who exceeded these limits were considered to have an unhealthy lifestyle. Regular physical activity was seen as a healthy behavior, which included ≥ 150 min of moderate physical activity, ≥ 75 min of vigorous physical activity, or ≥ 150 min of moderate-vigorous-intensity physical activity; at least 5 days a week of moderate physical activity; or vigorous exercise once a week [3]. For diet, we adopted the definition of an optimal intake of dietary components for cardiovascular health, which included the consumption of fruits, vegetables, fish, processed meats, and unprocessed meats. The target intakes for these items were based on a previous study [28]. A score of 1 was assigned for each ideal intake met, and a healthy diet was defined as the intake of at least four of these ideal dietary components [28]. We used five indicators to evaluate sleep quality, including sleep duration, chronotype, insomnia, snoring, and daytime dozing. Each healthy sleep factor was scored as 1, while unhealthy sleep factors were scored as 0. Good sleep quality was defined as a sleep score of ≥ 4 points [26]. Each lifestyle was scored as 1 if it was considered healthy and 0 if it was not. Thus, healthy lifestyles were associated with higher scores, ranging from 0 to 5. Finally, lifestyles were divided into unhealthy (0–1 score), general (2–3 score), and healthy (4–5 score) categories. Additional File 1: Table S1 provides specific details of lifestyle factors.

### Ascertainment of air pollution

Land Use Regression (LUR) models were developed from the European Study of Cohorts for Air Pollution Effects project to estimate annual average PM_2.5_, PM_10_, and PM_2.5−10_, NO_2_, and NO_x_ [29]. In this study, air pollution levels from 2010 were utilized as individual exposure levels. Subsequently, a LPA of the air pollution data was conducted to yield a new potential variable. This resulted in the identification of three latent levels that respectively represented comprehensive high, moderate, and low air pollution patterns, with detailed information provided in Additional File 1: Table S2 and Fig. S1.

### Assessment of outcome

The study outcome was all-cause mortality. Death certificates were obtained from the National Health Service (NHS) Information Centre (England and Wales) and the NHS Central Register (Scotland) [30]. Participants were followed from the time of enrollment in the UK Biobank Study until death or until March 2023, whichever occurred first.

### Assessment of covariates

According to existing studies [25, 29], several potential confounders were taken into account, including age (continuous), gender (male and female), ethnicity (white and non-white), education level (college or university degree and other), income (less than 31,000 € and greater than 31,000 €), BMI (18.5–24.9, < 18.5, 25.0-29.9, ≥ 30), depression (yes and no), dementia (yes and no), diabetes (yes and no), cancer (yes and no), cardiovascular disease (yes and no), and respiratory disease (yes and no).

### Statistical analyses

Study participants’ characteristics were presented as mean values with standard deviations (SD) for continuous variables and as percentages for categorical variables. LPA was employed to examine patterns of multiple air pollutants, with five latent profile models performed. The appropriate number of subgroups was determined based on a range of criteria including Aikaike’s Information Criterion (AIC), Bayesian Information Criterion (BIC), adjusted Bayesian Information Criterion (aBIC), Entropy, Lo-Mendell Rubin Likelihood Ratio Test (LMRT), and Bootstrap Likelihood Ratio Test (BLRT). A model was considered a better fit with smaller AIC, BIC, and aBIC values [31]. The larger the entropy value, the more accurate the class classification, with values ≥ 0.8 indicating a good profile solution [32]. Schoenfeld residuals confirmed the proportionality of hazards assumption, and Cox proportional hazards regression models were used to estimate hazard ratios (HRs) and 95% confidence intervals (CI). The modeled estimates were adjusted for factors such as age, gender, ethnicity, education level, income, BMI, depression, dementia, diabetes, cancer, cardiovascular disease, and respiratory disease. Missing variables were imputed using multiple imputation. Non-linear relationships were determined using restricted cubic spline curves with three knots to describe the relationship between air pollution and all-cause mortality.

To investigate the association between air pollution and lifestyle with all-cause mortality, hazard ratios and 95% confidence intervals were calculated. Meanwhile, lifestyle factors, including smoking, diet, alcohol consumption, physical activity, and sleep quality, and air pollution, including NO_2_, NO_x_, PM_2.5_, PM_10_, and PM_2.5−10_, were respectively investigated for their relationship with mortality risk.

We conducted a stratified analysis based on the levels of air pollution to examine the association between lifestyles and all-cause mortality across different air pollution subgroups. To assess the multiplicative interaction effect, we included interaction terms between air pollution (low, moderate, high) and lifestyles (unhealthy, general, healthy). Additionally, we further investigated the relationship between all-cause mortality and NO_2_, NO_x_, PM_2.5_, PM_10_, and PM_2.5−10_ among distinct lifestyle subgroups, with these five pollutants analyzed as continuous variables.

To assess the effect of grouping factors on the results, we carried out stratified analyses by age (< 60 and ≥ 60), gender (male and female), ethnicity (white and not-white), education level (college or university degree and other), income (less than 31,000 € and greater than 31,000 €).

To assess the robustness of our findings, we conducted five sensitivity analyses. Firstly, we excluded participants with missing covariate data. Secondly, we dichotomized each of the five categories of air pollution into high and low based on the median. Thirdly, we incorporated baseline BMI into the lifestyle variable. Fourthly, we excluded participants with a history of diabetes, cancer, cardiovascular disease, or respiratory disease at baseline. Fifthly, we excluded individuals who passed away during the initial three years of follow-up.

All statistical analyses were carried out with R software version 4.0.2 and Mplus version 7, and statistical significance (two-sided) was defined as *P* value < 0.05.

## Results

### Baseline characteristics of the participants

Table 1 presents the participant characteristics. Out of the 271,075 participants, 62,595 (23%) had an unhealthy lifestyle, 166,170 (61%) had a general lifestyle, and 42,310 (16%) had a healthy lifestyle. The proportion of low air pollution was 193,578 (71%), followed by 22,102 (8%) with moderate air pollution and 55,395 (21%) with high air pollution. For those with a healthy lifestyle, the mean age was 56.50 ± 8.33, 36.5% were male, 92.9% were White, 40.4% had a college or university degree, 51.0% had an income ≥ 31,000€, 39.9% had a normal BMI, 22.8% had cancer, 22.7% had cardiovascular disease, and 8.2% had respiratory disease. Among participants exposed to high air pollution, the mean age was 55.85 ± 8.24, 45.8% were male, 89.9% were White, 39.0% had a college or university degree, 46.5% had an income ≥ 31,000 €, 40.9% had a normal BMI, 22.6% had cancer, 29.4% had cardiovascular disease, and 8.8% had respiratory disease.

**Table 1:** Baseline characteristics of participants from UK Biobank according to lifestyle and air pollution (*n* = 271,075)

Characteristics	Lifestyle			Air pollution		
	Unhealthy lifestyle (*n* = 62,595)	General lifestyle (*n* = 166,170)	Healthy lifestyle (*n* = 42,310)	Low air pollution (*n* = 193,578)	Moderate air pollution(*n* = 22,102)	High air pollution(*n* = 55,395)
Age, mean(SD), y	57.20 (7.88)	56.81 (8.11)	56.50 (8.33)	57.14 (8.03)	56.82 (8.08)	55.85 (8.24)
Gender, n(%)						
Male	34,131 (54.5)	74,830 (45.0)	15,459 (36.5)	88,743 (45.8)	10,298 (46.6)	25,379 (45.8)
Female	28,464 (45.5)	91,340 (55.0)	26,851 (63.5)	104,835 (54.2)	11,804 (53.4)	30,016 (54.2)
Ethnicity, n(%)						
White	60,891 (97.3)	157,921 (95.0)	39,291 (92.9)	187,127 (96.7)	21,161 (95.7)	49,815 (89.9)
Not-white	1704 (2.7)	8249 (5.0)	3019 (7.1)	6451 (3.3)	941 (4.3)	5580 (10.1)
Education level, n(%)						
College or university degree	17,764 (28.4)	56,784 (34.2)	17,092 (40.4)	63,300 (32.7)	6745 (30.5)	21,595 (39.0)
Other	44,545 (71.2)	108,487 (65.3)	24,960 (59.0)	129,310 (66.8)	15,239 (68.9)	33,443 (60.4)
Missing data	286 (0.5)	899 (0.5)	258 (0.6)	968 (0.5)	118 (0.5)	357 (0.6)
Income (€), n (%)						
Less than 31,000	26,952 (43.1)	66,041 (39.7)	15,690 (37.1)	75,824 (39.2)	8788 (39.8)	24,071 (43.5)
Greater than 31,000	29,830 (47.7)	81,935 (49.3)	21,557 (51.0)	96,697 (50.0)	10,849 (49.1)	25,776 (46.5)
Missing data	5813 (9.3)	18,194 (10.9)	5063 (12.0)	21,057 (10.9)	2465 (11.2)	5548 (10.0)
BMI, n(%)						
18.5-24.9	15,573 (24.9)	55,277 (33.3)	18,493 (43.7)	63,935 (33.0)	6870 (31.1)	18,538 (33.5)
< 18.5	265 (0.4)	892 (0.5)	322 (0.8)	960 (0.5)	126 (0.6)	393 (0.7)
25.0-29.9	27,406 (43.8)	71,302 (42.9)	16,867 (39.9)	83,313 (43.0)	9614 (43.5)	22,648 (40.9)
≥ 30	19,351 (30.9)	38,699 (23.3)	6628 (15.7)	45,370 (23.4)	5492 (24.8)	13,816 (24.9)
Depression, n(%)	10,624 (17.0)	29,292(17.6)	7412 (17.5)	33,834 (17.5)	3681 (16.7)	9813 (17.7)
Missing data	900 (1.4)	2305 (1.4)	523 (1.2)	2667 (1.4)	293 (1.3)	768 (1.4)
Dementia, n(%)	1203 (1.9)	2847 (1.7)	660 (1.6)	3367 (1.7)	372 (1.7)	971 (1.8)
Diabetes, n(%)	4008 (6.4)	8008 (4.8)	1535 (3.6)	9249 (4.8)	1119 (5.1)	3183 (5.7)
Missing data	131 (0.2)	296 (0.2)	58 (0.1)	312 (0.2)	41 (0.2)	132 (0.2)
Cancer, n(%)	16,461 (26.3)	40,008 (24.1)	9649 (22.8)	48,254 (24.9)	5347 (24.2)	12,517 (22.6)
Cardiovascular disease^*^, n(%)	22,025 (35.2)	46,695 (28.1)	9606 (22.7)	55,610 (28.7)	6443 (29.2)	16,273 (29.4)
Missing data	76 (0.1)	172 (0.1)	49 (0.1)	203 (0.1)	24 (0.1)	70 (0.1)
Respiratory disease^#^, n(%)	5422 (8.7)	14,135 (8.5)	3460 (8.2)	16,220 (8.4)	1935 (8.8)	4862 (8.8)
Never smoking, n(%)	8033 (12.8)	99,757 (60.0)	39,809 (94.1)	107,677 (55.6)	12,196 (55.2)	27,726 (50.1)
Health diet, n(%)	744 (1.2)	15,489 (9.3)	15,760 (37.2)	22,432 (11.6)	2583 (11.7)	6978 (12.6)
No heavy alcohol consumption, n(%)	11,680 (18.7)	105,691 (63.6)	40,205 (95.0)	113,400 (58.6)	12,817 (58.0)	31,359 (56.6)
Adequate physical activity, n(%)	26,178 (41.8)	128,937 (77.6)	41,302 (97.6)	140,600 (72.6)	16,087 (72.8)	39,730 (71.7)
Good sleep quality, n(%)	4388 (7.0)	62,553 (37.6)	36,887 (87.2)	74,800 (38.6)	8413 (38.1)	20,615 (37.2)
NO_2,_ mean(SD)	26.78 (7.66)	26.26 (7.55)	25.86 (7.53)	23.65 (5.24)	24.29 (6.47)	36.45 (6.22)
NO_x,_ mean(SD)	44.39 (15.83)	43.28 (15.32)	42.42(15.14)	37.86 (8.86)	40.11 (12.55)	64.08 (16.97)
PM_10,_ mean(SD)	16.25 (1.89)	16.19 (1.91)	16.13 (1.91)	15.40 (1.26)	20.04 (1.37)	17.43 (1.34)
PM_2.5,_ mean(SD)	10.03 (1.08)	9.94 (1.04)	9.87 (1.03)	9.57 (0.73)	9.95 (1.14)	11.28 (0.89)
PM_2.5−10,_ mean(SD)	6.43 (0.90)	6.42 (0.90)	6.40 (0.90)	6.05 (0.38)	8.87 (0.45)	6.72 (0.66)

Abbreviations: SD, standard deviation; BMI, body mass index; NO_2_, nitrogen dioxide; NOx, nitrogen oxides; PM_2.5_, particulate matter with diameter ≤ 2.5 μm; PM_10_, particulate matter with diameter ≤ 10 μm; PM_2.5−10_, particulate matter with diameter 2.5–10 μm

^*^ Cardiovascular disease: heart attack, angina, stroke, and high blood pressure

^#^ Respiratory disease: asthma, chronic obstructive airways disease, emphysema/chronic bronchitis, bronchiectasis, interstitial lung disease, other respiratory problems

### Associations of lifestyle with all-cause mortality

The median follow-up duration was 13.9 years, and 21,602 participants died during the study period. The findings in Table 2 show people with unhealthy lifestyles have a significantly enhanced risk of death compared to people with general lifestyles before and after covariate adjustment (HR = 1.513, 95% CI, 1.469–1.558; HR = 1.315, 95% CI, 1.277–1.355), while people with healthy lifestyles have a significantly lower risk of death (HR = 0.735, 95% CI, 0.703–0.768; HR = 0.821, 95% CI, 0.785–0.858). Upon analyzing the five lifestyle variables separately, the results indicated that, after accounting for covariates, never smoking (HR = 0.690, 95% CI, 0.671–0.710), a healthy diet (HR = 0.924, 95% CI, 0.886–0.964), adequate physical activity (HR = 0.773, 95% CI, 0.751–0.795), and good sleep quality (HR = 0.894, 95% CI, 0.869–0.920) were all significantly correlated with all-cause mortality.

**Table 2:** Associations between lifestyle and all cause mortality

Variables	HR(95%CI) from Model 1	*P*	HR(95%CI) from Model 2	*P*
Lifestyle factors				
Never smoking	0.527(0.514–0.542)	< 0.001	0.690(0.671–0.710)	< 0.001
Health diet	0.949(0.911–0.991)	0.020	0.924(0.886–0.964)	< 0.001
No heavy alcohol consumption	1.007(0.980–1.034)	0.600	0.977(0.950–1.004)	0.090
Adequate physical activity	0.710(0.691–0.730)	< 0.001	0.773(0.751–0.795)	< 0.001
Good sleep quality	0.803(0.781–0.826)	< 0.001	0.894(0.869–0.920)	< 0.001
Lifestyle				
General lifestyle	Ref.		Ref.	
Unhealthy lifestyle	1.513(1.469–1.558)	< 0.001	1.315(1.277–1.355)	< 0.001
Healthy lifestyle	0.735(0.703–0.768)	< 0.001	0.821(0.785–0.858)	< 0.001

Model 1: Crude

Model 2: Adjusted for age, gender, ethnicity, education level, income, BMI, depression, dementia, diabetes, cancer, cardiovascular disease, and respiratory disease

### Associations of air pollution with all-cause mortality

Table 3 illustrates that high air pollution increased the risk of all-cause mortality. Specifically, compared to low air pollution, the difference between moderate air pollution and mortality was not statistically significant before and after covariate adjustment (HR = 0.981, 95% CI, 0.933–1.031; HR = 0.993, 95% CI, 0.945–1.044). Conversely, after adjusting for covariates, individuals exposed to high air pollution were independently associated with a higher risk of death compared to those exposed to low air pollution (HR = 1.162, 95% CI, 1.124–1.201). Similarly, the five indicators of air pollution were analyzed separately, revealing a significant association between all-cause mortality and NO_2_ (HR = 1.010, 95% CI, 1.008–1.012), NO_x_ (HR = 1.005, 95% CI, 1.004–1.006), PM_2.5_ (HR = 1.069, 95% CI, 1.056–1.083), and PM_10_ (HR = 1.017, 95% CI, 1.010–1.024) after adjustments for covariates. Additionally, a multiple-adjusted restricted cubic spline with three knots was used to describe the relationship between air pollution and all-cause mortality. The findings indicated NO_x_, PM_10_, and PM_2.5−10_ had a nonlinear relationship with all-cause mortality (*p* for nonlinearity < 0.05), whereas NO_2_ and PM_2.5_ exhibited an approximately linear distribution with all-cause mortality (*p* for nonlinearity > 0.05) (Fig. S2).

### Interaction analysis of lifestyle and air pollution with all-cause mortality

No statistically significant interaction was identified between lifestyle and air pollution concerning all-cause mortality (*P* for interaction > 0.05; as illustrated in Table 4). In the air pollution subgroup, a healthy lifestyle was associated with a lower risk of all-cause mortality compared to a general lifestyle. For instance, in areas with high air pollution levels, individuals with healthy lifestyles had a lower mortality risk (HR = 0.772, 95% CI, 0.697–0.856) than those with general lifestyles; similar results were observed in areas with moderate (HR = 0.710, 95% CI, 0.602–0.838) and low (HR = 0.852, 95% CI, 0.809–0.897) air pollution. Conversely, an unhealthy lifestyle was associated with a higher risk of all-cause mortality across all air pollution levels.

**Table 3:** Associations between air pollution and all cause mortality

Variables	HR (95% CI) from Model 1	*P*	HR (95% CI) from Model 2	*P*
Air pollution				
NO_2_	1.007(1.005–1.008)	< 0.001	1.010(1.008–1.012)	< 0.001
NOx	1.004(1.003–1.005)	< 0.001	1.005(1.004–1.006)	< 0.001
PM_2.5_	1.063(1.050–1.076)	< 0.001	1.069(1.056–1.083)	< 0.001
PM_10_	1.015(1.008–1.022)	< 0.001	1.017(1.010–1.024)	< 0.001
PM_2.5−10_	1.007(0.992–1.022)	0.400	1.014(0.999–1.029)	0.064
Air pollution levels				
Low air pollution	Ref.		Ref.	
Moderate air pollution	0.981(0.933–1.031)	0.447	0.993(0.945–1.044)	0.789
High air pollution	1.083(1.048–1.119)	< 0.001	1.162(1.124–1.201)	< 0.001

Model 1: Crude

Model 2: Adjusted for age, gender, ethnicity, education level, income, BMI, depression, dementia, diabetes, cancer, cardiovascular disease, respiratory disease, and lifestyles

Abbreviations: NO_2_, nitrogen dioxide; NOx, nitrogen oxides; PM_2.5_, fine particulate matter with diameter ≤ 2.5 μm; PM_10_, particulate matter with diameter ≤ 10 μm; PM_2.5−10_, particulate matter with diameter 2.5–10 μm HR of NO2, NOx, PM2.5, PM10, and PM2.5-10 was evaluated by per 1µg/m³ increase

**Table 4:** Associations of lifestyle with all cause mortality by air pollution

Variables	No of participants	HR (95% CI)	*P*
High air pollution			
General lifestyle	33,617	Ref.	
Unhealthy lifestyle	13,903	1.239(1.163-1.319)	<0.001
Healthy lifestyle	7875	0.772(0.697-0.856)	<0.001
Moderate air pollution			
General lifestyle	13,659	Ref.	
Unhealthy lifestyle	5016	1.310(1.178-1.457)	<0.001
Healthy lifestyle	3427	0.710(0.602-0.838)	<0.001
Low air pollution			
General lifestyle	118,894	Ref.	
Unhealthy lifestyle	43,676	1.333(1.287-1.382)	<0.001
Healthy lifestyle	31,008	0.852(0.809-0.897)	<0.001

All models were adjusted for age, gender, ethnicity, education level, income, BMI, depression, dementia, diabetes, cancer, cardiovascular disease, and respiratory disease

An examination of the connection between air pollution and all-cause mortality within various lifestyle subgroups, as presented in Table 5, revealed that the association between the five types of air pollution and all-cause mortality failed to yield significant results within the healthy lifestyle group (*p* > 0.05). Conversely, the relationship between the five types of air pollution and all-cause mortality was statistically significant for both general and unhealthy lifestyles. Additionally, the correlation between PM_2.5_ and mortality weakened as lifestyles became healthier, although this difference was deemed insignificant within the realm of healthy lifestyles.

**Table 5:** Associations of air pollution with all cause mortality by lifestyle

Variables	HR (95% CI)	*P*
Healthy lifestyle		
NO_2_	1.002(0.996–1.007)	0.602
NOx	1.000(0.997–1.003)	0.972
PM_2.5_	1.012(0.972–1.053)	0.571
PM_10_	0.982(0.961–1.003)	0.096
PM_2.5−10_	0.958(0.915–1.004)	0.073
General lifestyle		
NO_2_	1.011(1.009–1.013)	< 0.001
NOx	1.005(1.004–1.006)	< 0.001
PM_2.5_	1.079(1.061–1.097)	< 0.001
PM_10_	1.024(1.014–1.033)	< 0.001
PM_2.5−10_	1.025(1.006–1.045)	0.011
Unhealthy lifestyle		
NO_2_	1.010(1.007–1.013)	< 0.001
NOx	1.005(1.003–1.006)	< 0.001
PM_2.5_	1.067(1.044–1.090)	< 0.001
PM_10_	1.017(1.005–1.030)	< 0.001
PM_2.5−10_	1.014(0.988–1.040)	< 0.001

All models were adjusted for age, gender, ethnicity, education level, income, BMI, depression, dementia, diabetes, cancer, cardiovascular disease, and respiratory disease

### Subgroup and sensitivity analysis

The stratified analysis, based on age, gender, ethnicity, education level, and income, was conducted, and the results are presented in Additional File 1: Table S3. The analysis revealed that the relationship between lifestyle and all-cause mortality was more pronounced in males and younger individuals in both cohorts, with a significant interaction (*P* < 0.02). Furthermore, Additional File 1: Table S4 indicated that air pollution and all-cause mortality did not notably vary across subgroups. Overall, the subgroup analyses were consistent with the main analysis. Additionally, we conducted five sensitivity analyses. All associations remained significant and consistent with the overall study findings, indicating the robustness of our results, as presented in Additional File 1: Table S5-S8.

## Discussion

In this large cohort of more than 270,000 participants, we found that, in terms of lifestyles, healthy lifestyles were associated with a reduced risk of all-cause mortality, in contrast to general lifestyles, while unhealthy lifestyles were significantly associated with an increased risk of all-cause mortality. In terms of air pollution, high air pollution exposure, including NO_2_, NO_x_, PM_2.5_, and PM_10_, was positively associated with the risk of all-cause mortality. Moreover, we found that the strength of the association between unhealthy lifestyles and mortality risk varied across different air pollutant subgroups, with stronger associations observed in subgroups with lower levels of air pollution. Finally, a range of subgroup and sensitivity analyses reinforced the robustness of our findings.

Consistent with previous studies [17, 33–36], our study reveals a significant correlation between lifestyle and all-cause mortality. Factors that contribute to protecting against mortality include never smoking, a healthy diet, sufficient physical activity, and good sleep quality, all of which have been confirmed by multiple studies [25, 37–39]. Smoking exhibits the strongest association with mortality, likely attributable to the nicotine it produces, which elevates inflammation and stimulates oxidative stress. In our study, alcohol consumption did not show a significant relationship with all-cause mortality, consistent with Zhang et al. [20]. However, one study observed that light to moderate drinkers demonstrated a healthier lifestyle than non-drinkers [40]. Furthermore, research suggests wine contains biologically active compounds such as anthocyanins and resveratrol that might regulate lipid metabolism, reduce oxidative stress, and mitigate against cancer [41]. Given these conflicting findings, the relationship between alcohol consumption and the risk of death merits further confirmation. Our study suggests that, in addition to never smoking, a healthy lifestyle is more protective against mortality than just considering lifestyle factors, because multiple lifestyle factors may have a synergistic effect. Consequently, we believe it is essential to emphasize maintaining a variety of healthy lifestyles when promoting health.

Our study has revealed a significant correlation between high levels of atmospheric pollution and all-cause mortality. The relationship between NO_2_, NO_x_, PM_2.5_, PM_10_, and all-cause mortality was statistically significant, consistent with previous research findings [42–45]. Notably, PM_2.5_ was found to have the strongest association with mortality, which is consistent with prior estimates [15, 46]. This robust association can be attributed to two primary factors. Firstly, the small size of PM_2.5_ particles means that they can remain suspended in the atmosphere for protracted periods of time and hence increase the likelihood of causing inhalation damage to the lungs. Secondly, due to the small size of particles within PM_2.5_, they are capable of absorbing toxic substances in the air before penetrating deeply into the lungs [47]. Furthermore, our findings also suggest that PM_10_, containing primarily natural elements instead of heavy metals, has relatively less toxicity due to its small total surface area [48]. It is noteworthy, however, that our study demonstrated that the mortality risk associated with combined exposure to multiple air pollutants is stronger than that of individual exposure. Nonetheless, the exact mechanism underpinning the relationship between exposure to mixed air pollutants and mortality is not yet fully understood. We conjecture that synergistic or additive effects may occur when exposed to multiple air pollutants [49], resulting in more severe respiratory tract damage and inflammatory responses than from individual exposure alone.

We discovered that the correlation between lifestyle and mortality risk remained robust across various subgroups of air pollutants. After categorizing by lifestyle, the associations between the five air pollutants and all-cause mortality were insignificant in the healthy lifestyle group. Conversely, in the other two lifestyle groups, significant associations were observed between the air pollutants and all-cause mortality. Thus, we speculate that individuals leading a healthier lifestyle may have relatively stringent requirements for their residential and work environments and may be more mindful of their exposure to harmful pollutants in their daily lives. Furthermore, subgroup analyses indicated that the protective impact of a healthy lifestyle on mortality risk was more evident among individuals under 60 years of age and in males. Various explanations may account for this possibility. Firstly, older individuals often have more underlying medical conditions, and thus the effect of an improved lifestyle may be less impactful than in younger individuals. Therefore, the protective influence of a healthy lifestyle on the risk of death in individuals under 60 years of age will be more conspicuous. Secondly, our analysis revealed that among the five lifestyle factors, the protective impact of non-smoking was the most robust, and in reality, a higher prevalence of smoking is found in males than females, while males are more active than females. Thus, adopting a healthier lifestyle, such as quitting smoking and engaging in more physical activity, will render the relationship between lifestyle and death risk more pronounced in males.

To the best of our knowledge, few studies have used LPA to categorize air pollutants with the aim of investigating the association between lifestyle, air pollution, and the risk of all-cause mortality, as well as exploring the interactions between lifestyle and air pollution. Furthermore, this study had a large sample size, longitudinal follow-up, rigorously defined variables, and the different subgroups and characteristics of the analysis have consolidated our findings. However, there are several limitations to consider. Firstly, lifestyle factors were self-reported, and thus, measurement errors may be unavoidable. Secondly, while we included five different lifestyles in our primary analysis, with BMI added in our sensitivity analysis, there may be additional behavioral factors that can potentially impact the results but were not taken into consideration. Thirdly, some previous studies suggest that exposure to ozone, carbon monoxide, and sulfur dioxide is linked to an increased risk of mortality [46, 50, 51]; however, such data was not available in the UK Biobank study. Fourthly, we used average air pollution concentrations from 2010 for our analysis and did not account for changes in pollution levels over time, though prior research indicates that air pollution levels have remained relatively stable during the period studied by the UK Biobank [52]. Lastly, while we made adjustments for various potential confounders, residual confounders from unmeasured or unknown variables may still have an impact on our analysis.

## Conclusions

Unhealthy lifestyles and exposure to air pollution were significantly associated with an increased risk of all-cause mortality, while healthy lifestyles significantly reduced the risk of mortality. Furthermore, there exists a potential cumulative impact of several air pollutants on mortality. As the density of air pollution decreased from high to low, the interrelation between unhealthy lifestyles and mortality risk became more significant. These discoveries underscore the significance of coordinated measures to enhance air quality and adopt a healthy lifestyle to minimize the likelihood of death.

### Electronic supplementary material

Below is the link to the electronic supplementary material.

Supplementary Material 1

Supplementary Material 2

Supplementary Material 3

## Data Availability

No datasets were generated or analysed during the current study.

## Abbreviations

PM: Particle matter
NO_2_: Nitrogen dioxide
NO_x_: Nitrogen oxides
LPA: Latent profile analysis
BMI: Body mass index
LUR: Land use regression
NHS: National health service
SD: Standard deviations
AIC: Aikaike’s information criterion
BIC: Bayesian information criterion
aBIC: Adjusted bayesian information criterion
LMRT: Lo-mendell rubin likelihood ratio test
BLRT: Bootstrap likelihood ratio test
HRs: Hazard ratios
Cis: Confidence interval
