# Supplementary figures and images for: Health impacts of lifestyle and ambient air pollution patterns on all-cause mortality: a UK Biobank cohort study
Source: BMC Public Health. 2024 Jun 25;24:1696. doi: 10.1186/s12889-024-19183-5 (PMC11202323; doi:10.1186/s12889-024-19183-5)

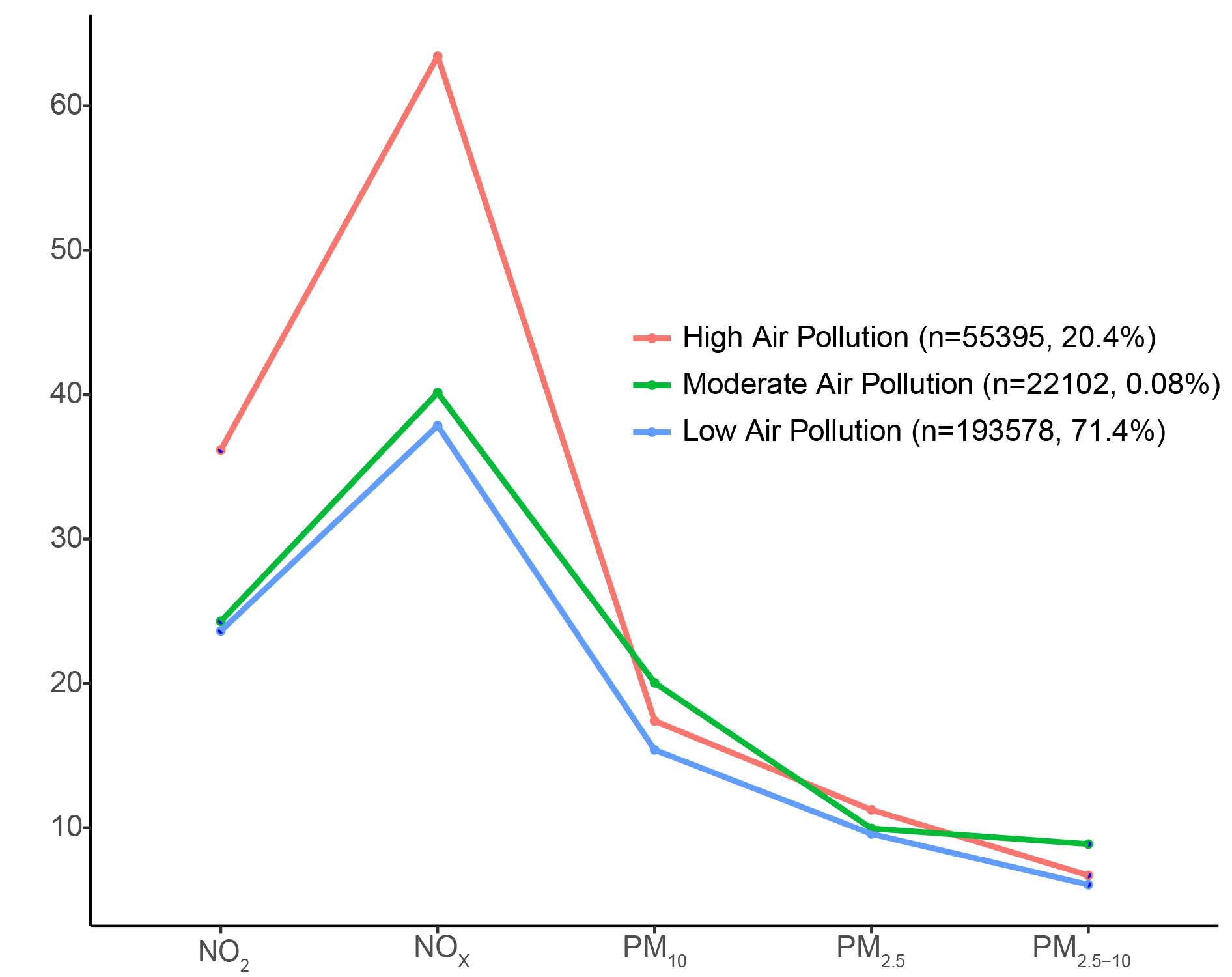

Supplement: Supplementary file 1 — Supplementary Material 1 [file 12889_2024_19183_MOESM1_ESM.tif]
