# Supplementary material for: Health impacts of lifestyle and ambient air pollution patterns on all-cause mortality: a UK Biobank cohort study
Source: BMC Public Health. 2024 Jun 25;24:1696. doi: 10.1186/s12889-024-19183-5 (PMC11202323; doi:10.1186/s12889-024-19183-5)
Supplement: Supplementary file 3 — Supplementary Material 3 [file 12889_2024_19183_MOESM3_ESM.docx]

Table S1 Definition and composition of lifestyle factors.

| Lifestyle factors | Definition of factors | Classification of lifestyle status | | UK Biobank field code |
| --- | --- | --- | --- | --- |
|  |  | healthy lifestyle | unhealthy lifestyle |  |
| Smoking |  | never smoker | current/previous smoker | 20116 |
| Alcohol consumption | Pure alcohol (g/100 ml): red wine-13; white wine-12; beer-4.5; spirits-40; fortied wine-18  Pure alcohol intake (g/day):  (red wine×13/100×125+white wine×12/100×125+ beer×4.5/100×568.3+spirits×40/100×25+ fortified wine×18/100×75)/7 | never;  0<female≤20g/day;  0<male≤30g/day | female>20g/day;  male>30g/day; | 20117, 1558, 1568, 1578, 1588, 1598, 1608 |
| Physical activity | 884-In a typical week, on how many days did you do 10 minutes or more of moderate physical activities like carrying light loads, cycling at normal pace? (Do not include walking)  894-How many minutes did you usually spend doing moderate activities on a typical day?  904-In a typical week, how many days did you do 10 minutes or more of vigorous physical activity? (These are activities that make you sweat or breathe hard such as fast cycling, aerobics, heavy lifting)  914-How many minutes did you usually spend doing vigorous activities on a typical day? | ≥150 min of moderate physical activity;  or ≥75 min of vigorous physical exercise;  or ≥150 min of combined moderate and vigorous physical activity;  or moderate physical activity at least 5 days a week; or vigorous activity once a week | 0<150 min of moderate physical activity;  or 0<75 min of vigorous physical exercise;  or 0<150 min of combined moderate and vigorous physical activity; | 884, 894, 904, 914 |
| Diet* | Fruit ≥3 servings/day;  Vegetable ≥3 servings/day;  Fish ≥2 servings/week;  Processed meats ≤1 serving/week;  Unprocessed meats ≤2 serving/week | ≥ four points | < four points | 1309, 1319, 1289, 1299, 1329, 1339, 1349, 1369, 1379, 1389 |
| Sleep quality^#^ | 1180-Morning/evening person (chronotype) Do you consider yourself to be?  1160-About how many hours sleep do you get in every 24 hours? (please include naps)  1200-Do you have trouble falling asleep at night or do you wake up in the middle of the night?  1210-Does your partner or a close relative or friend complain about your snoring?  1220-How likely are you to doze off or fall asleep during the daytime when you don't mean to? (e.g. when working, reading or driving) | ≥ four points | < four points | 1180, 1160, 1200, 1210, 1220 |

* Diet: If participants achieved the intake goal, they were considered adequate intake of the diet component and received one point out of five.

^#^ Sleep quality: A healthy sleep involved sleep duration (7–8h/day), chronotype (definitely a “morning” person more a “morning” than “evening” person), insomnia symptoms (never/rarely), snoring (no), and daytime dozing (never/rarely), participants received one point for each factor if requirements were met above, five points in total.

Table S2 Comparison of LPA models with different latent classes based on model selection statistics.

|  | AIC | BIC | aBIC | Entropy | LMRT  *P*-value | BLRT  *P*-value |
| --- | --- | --- | --- | --- | --- | --- |
| One-class | 6746256.97 | 6746362.08 | 6746330.30 | - | - | - |
| Two-class | 6359929.21 | 6360097.38 | 6360046.53 | 0.967 | <0.001 | <0.001 |
| **Three-class** | **6074338.34** | **6074569.56** | **6074499.64** | **0.915** | **<0.001** | **<0.001** |
| Four-class | 5789357.36 | 5789651.65 | 5789562.66 | 0.905 | <0.001 | <0.001 |
| Five-class | 5637459.48 | 5637816.83 | 5637708.77 | 0.910 | <0.001 | <0.001 |

Notes: AIC Akaike’s information criterion, BIC Bayesian information criterion, LMRT Lo-mendell rubin likelihood ratio test, BLRT Bootstrap likelihood ratio test.

Table S3 Subgroup analyses for associations of lifestyle with all cause mortality.

| Subgroups | Unhealthy lifestyle | General lifestyle | Healthy lifestyle | HR (95% CI) | *P* value for interaction |
| --- | --- | --- | --- | --- | --- |
| Age |  |  |  |  | <0.001 |
| <60 | 33909(54.2%) | 92709(55.8%) | 23974(56.7%) | 0.62(0.60-0.65) |  |
| ≥60 | 28686(45.8%) | 73461(44.2%) | 18336(43.3%) | 0.72(0.70-0.74) |  |
| Gender |  |  |  |  | 0.018 |
| Male | 34131(54.5%) | 74830(45.0%) | 15459(36.5%) | 0.69(0.67-0.71) |  |
| Female | 28464(45.5%) | 91340(55.0%) | 26851(63.5%) | 0.77(0.75-0.80) |  |
| Ethnicity |  |  |  |  | 0.234 |
| White | 60891(97.3%) | 157921(95.0%) | 39291(92.9%) | 0.69(0.67-0.70) |  |
| Not-white | 1704(2.7%) | 8249(5.0%) | 3019(7.1%) | 0.78(0.69-0.89) |  |
| Education level |  |  |  |  | 0.004 |
| College or university degree | 17826(28.5%%) | 56980(34.4%) | 17162(40.6%) | 0.75(0.71-0.78) |  |
| Other | 44769(71.5%) | 109190(65.7%) | 25148(59.4%) | 0.69(0.67-0.70) |  |
| Income |  |  |  |  | 0.098 |
| Less than 31,000 | 30380(48.5%) | 76463(46.0%) | 18501(43.7%) | 0.69(0.67-0.71) |  |
| Greater than 31,000 | 32215(51.5%) | 89707(54.0%) | 145731(56.3%) | 0.71(0.68-0.74) |  |

Table S4 Subgroup analyses for associations of air pollution with all cause mortality.

| Subgroups | Low air pollution | Moderate air pollution | High air pollution | HR (95% CI) | *P* value for interaction |
| --- | --- | --- | --- | --- | --- |
| Age |  |  |  |  | 0.435 |
| <60 | 104725(54.1%) | 12282(55.6%) | 33585(60.6%) | 1.09(1.06-1.13) |  |
| ≥60 | 88853(45.9%) | 9820(44.4%) | 21810(39.4%) | 1.07(1.05-1.10) |  |
| Gender |  |  |  |  | 0.230 |
| Male | 88743(45.8%) | 10298(46.6%) | 25379(45.8%) | 1.05(1.02-1.07) |  |
| Female | 104835(54.2%) | 11804(53.4%) | 30016(54.2%) | 1.02(1.00-1.05) |  |
| Ethnicity |  |  |  |  | 0.270 |
| White | 18727(96.7%) | 21161(95.7%) | 49815(89.9%) | 1.05(1.03-1.07) |  |
| Not-white | 6451(3.3%) | 941(4.3%) | 5580(10.1%) | 1.08(1.00-1.16) |  |
| Education level |  |  |  |  | 0.773 |
| College or university degree | 63502(32.8%) | 6770(30.6%) | 21696(39.2%) | 1.00(0.96-1.03) |  |
| Other | 130076(67.2%) | 15332(69.4%) | 33699(60.8%) | 1.07(1.05-1.09) |  |
| Income |  |  |  |  | 0.207 |
| Less than 31,000 | 87661(45.3%) | 10203(46.2%) | 27480(49.6%) | 1.03(1.01-1.05) |  |
| Greater than 31,000 | 105917(54.7%) | 11899(53.8%) | 27915(50.4%) | 1.00(0.97-1.03) |  |

Table S5 Sensitivity analysis for associations between lifestyle and air pollution and mortality after removing the missing value.

| Variables | HR(95%CI) from Model 1 | *P* | HR(95%CI) from Model 2 | *P* |
| --- | --- | --- | --- | --- |
| Lifestyle factors |  |  |  |  |
| Never smoking | 0.528(0.513-0.544) | <0.001 | 0.692(0.672-0.713) | <0.001 |
| Health diet | 0.944(0.902-0.989) | 0.014 | 0.922(0.880-0.965) | <0.001 |
| No heavy alcohol consumption | 0.997(0.969-1.026) | 0.846 | 0.974(0.946-1.004) | 0.087 |
| Adequate physical activity | 0.707(0.686-0.729) | <0.001 | 0.773(0.749-0.797) | <0.001 |
| Good sleep quality | 0.798(0.774-0.822) | <0.001 | 0.893(0.866-0.921) | <0.001 |
| Lifestyle |  |  |  |  |
| General lifestyle | Ref. |  | Ref. |  |
| Unhealthy lifestyle | 1.520(1.473-1.569) | <0.001 | 1.315(1.274-1.358) | <0.001 |
| Healthy lifestyle | 0.731(0.696-0.767) | <0.001 | 0.826(0.787-0.867) | <0.001 |
| Air pollution |  |  |  |  |
| NO_2_ | 1.007(1.005-1.008) | <0.001 | 1.010(1.008-1.012) | <0.001 |
| NOx | 1.004(1.003-1.005) | <0.001 | 1.004(1.003-1.005) | <0.001 |
| PM_2.5_ | 1.061(1.047-1.075) | <0.001 | 1.069(1.054-1.083) | <0.001 |
| PM_10_ | 1.014(1.007-1.022) | <0.001 | 1.017(1.010-1.025) | <0.001 |
| PM_2.5-10_ | 1.007(0.991-1.023) | 0.413 | 1.014(0.998-1.030) | 0.083 |
| Air pollution levels |  |  |  |  |
| Low air pollution | Ref. |  | Ref. |  |
| Moderate air pollution | 0.991(0.939-1.045) | 0.727 | 1.008(0.955-1.063) | 0.782 |
| High air pollution | 1.076(1.039-1.114) | <0.001 | 1.157(1.117-1.199) | <0.001 |

Model 1: Crude.

Model 2: Lifestyle factors and lifestyle were adjusted for age, gender, ethnicity, education level, income, BMI, depression, dementia, diabetes, cancer, cardiovascular disease, and respiratory disease. Air pollution and Air pollution levels were adjusted for age, gender, ethnicity, education level, average total household income before tax, BMI, depression, dementia, diabetes, cancer, cardiovascular disease, respiratory disease, and lifestyles. Abbreviations: NO_2_, nitrogen dioxide; NOx, nitrogen oxides; PM_2.5_, fine particulate matter with diameter ≤2.5 μm; PM_10_, particulate matter with diameter ≤10 μm; PM_2.5-10_, particulate matter with diameter 2.5-10 μm.

Table S6 Sensitivity analysis for associations between air pollution (median) and all-cause mortality.

| Subgroups | HR (95% CI) |
| --- | --- |
| NO_2_ |  |
| Low NO_2_ | Ref. |
| High NO_2_ | 1.119(1.089-1.150) |
| NO_X_ |  |
| Low NO_X_ | Ref. |
| High NO_X_ | 1.131(1.101-1.162) |
| PM_10_ |  |
| Low PM_10_ | Ref. |
| High PM_10_ | 1.078(1.050-1.107) |
| PM_2.5_ |  |
| Low PM_2.5_ | Ref. |
| High PM_2.5_ | 1.106(1.076-1.136) |
| PM_2.5-10_ |  |
| Low PM_2.5-10_ | Ref. |
| High PM_2.5-10_ | 1.055(1.027-1.083) |

All models were adjusted for age, gender, ethnicity, education level, income, BMI, depression, dementia, diabetes, cancer, cardiovascular disease, respiratory disease, smoking, diet, alcohol consumption, physical activity, and sleep.

Abbreviations: NO_2_, nitrogen dioxide; NOx, nitrogen oxides; PM_2.5_, fine particulate matter with diameter ≤2.5 μm; PM_10_, particulate matter with diameter ≤10 μm; PM_2.5-10_, particulate matter with diameter 2.5-10 μm.

Table S7 Sensitivity analysis for associations between lifestyle (including BMI) and all-cause mortality.

| Variables | HR(95%CI) from Model 1 | *P* | HR(95%CI) from Model 2 | *P* |
| --- | --- | --- | --- | --- |
| BMI |  |  |  |  |
| Abnormal BMI | Ref. |  | Ref. |  |
| Normal BMI | 0.706(0.685-0.728) | <0.001 | 0.958(0.928-0.988) | 0.007 |
| Lifestyle |  |  |  |  |
| General lifestyle | Ref. |  | Ref. |  |
| Unhealthy lifestyle | 1.565(1.522-1.609) | <0.001 | 1.304(1.267-1.341) | <0.001 |
| Healthy lifestyle | 0.715(0.670-0.764) | <0.001 | 0.831(0.778-0.888) | <0.001 |

Model 1: Crude.

Model 2: Adjusted for age, gender, ethnicity, education level, income, depression, dementia, diabetes, cancer, cardiovascular disease, and respiratory disease.

The lifestyle consisted of never smoking, no heavy alcohol consumption, adequate physical activity, health diet, good sleep quality, and abnormal BMI.

Table S8 Sensitivity analysis after excluding individuals.

| Variables | HR(95%CI) | *P* |
| --- | --- | --- |
| Excluding individuals with diabetes, cancer, cardiovascular disease, and respiratory disease* (N=128,444) |  |  |
| Lifestyle |  |  |
| General lifestyle | Ref. |  |
| Unhealthy lifestyle | 1.261(1.163-1.368) | <0.001 |
| Healthy lifestyle | 0.912(0.823-1.011) | 0.078 |
| Air pollution |  |  |
| Low air pollution | Ref. |  |
| Moderate air pollution | 1.041(0.916-1.183) | 0.536 |
| High air pollution | 1.132(1.037-1.234) | <0.001 |
| Excluding events that occurred within the first three years of follow-up ^#^(N=268,907) |  |  |
| Lifestyle |  |  |
| General lifestyle | Ref. |  |
| Unhealthy lifestyle | 1.291(1.251-1.332) | <0.001 |
| Healthy lifestyle | 0.832(0.794-0.871) | <0.001 |
| Air pollution |  |  |
| Low air pollution | Ref. |  |
| Moderate air pollution | 0.989(0.939-1.043) | 0.691 |
| High air pollution | 1.151(1.112-1.192) | <0.001 |

*Lifestyle controlled for age, gender, ethnicity, education level, income, BMI, depression, and dementia. Air pollution levels controlled for age, gender, ethnicity, education level, income, BMI, depression, dementia, and lifestyles.

^#^Lifestyle controlled for age, gender, ethnicity, education level, income, BMI, depression, dementia, diabetes, cancer, cardiovascular disease, and respiratory disease. Air pollution with mortality by controlled for age, gender, ethnicity, education level, income, BMI, depression, dementia, diabetes, cancer, cardiovascular disease, respiratory disease, and lifestyles.
